# Minimising infection risks: Safe laundry practices in UK care homes through research and guidance development

**DOI:** 10.1371/journal.pgph.0006417

**Published:** 2026-05-13

**Authors:** Sapphire Crosby, Janet McMahon, Joy Allen, Jackie Hook, Kathryn Hinsliff-Smith, Katie Laird

**Affiliations:** 1 Faculty of Health and Life Sciences, De Montfort University, Leicester, United Kingdom; 2 Cheshire and Wirral Partnership NHS, Warrington, United Kingdom; 3 Kirklees Council, Huddersfield, United Kingdom; 4 Independent Laundry Technical Consultant, West Yorkshire, United Kingdom; 5 Leicester School of Nursing and Midwifery, De Montfort University, Leicester, United Kingdom; 6 School of Pharmacy, De Montfort University, Leicester, United Kingdom; ICDDRB: International Centre for Diarrhoeal Disease Research Bangladesh, BANGLADESH

## Abstract

Those living in care homes are often the most vulnerable in our society, either due to their age, frailty or ongoing physical and mental health needs. Care homes are more susceptible to infectious diseases due to their communal living environments, resulting in transmission of infections through day-to-day interactions. Infection prevention and control (IPC) is therefore a priority for all care homes, including safe practices for laundry management. Despite the clear risk posed by contaminated textiles, the implementation and understanding of effective laundry IPC measures in care homes remains underexplored. This study aimed to assess current laundry management practices, awareness of IPC guidance, and the barriers care homes face in adopting standardised laundering protocols. Using a mixed-methods approach, data were gathered from 1,062 online survey responses, follow-up interviews, and focus groups with care home managers, staff, and industry experts. Key findings revealed a significant lack of awareness and inconsistent application of the national Health Technical Memorandum (HTM-01–04). Over 50% of care homes lacked formal management of laundry training programs, and only 46% of care home staff reported having clear IPC procedures for linen management. Additional challenges included spatial constraints, costs, equipment maintenance, and the difficulty of laundering mixed textiles, such as residents’ personal clothing, at appropriate disinfection temperatures without damage. The survey also showed that nearly half of the managers expressed a preference for the outsourcing of laundry to commercial laundries. The findings highlight an urgent need for accessible, practical, and sector-specific linen IPC guidance, tailored to the unique care home environments. In response, adapted guidance and educational resources were co-created with sector stakeholders to support implementation. This study is the first of its kind in the UK and emphasises that safe linen management is fundamental to infection control in care homes, protecting both vulnerable residents and staff from healthcare-associated infections.

## Introduction‌‌

Infection prevention and control (IPC) is essential for health management within care homes for older people since residents are often vulnerable due to their age, comorbidities, or compromised immunity. The type of aged care homes referred to in this study are for those aged over 65, providing permanent living arrangements, health and social provision in private and communal living spaces. These types of environments, often shared, where the movement and management of materials, including linen, form an integral part of the day‑to‑day operations of a care home, all under the auspices of IPC policies and practices. This makes the handling of linen a critical yet sometimes overlooked component of IPC protocols [1], where poor or improper linen practices can facilitate the spread of healthcare-associated infections (HCAIs), directly impacting residents and care home staff. Amongst various IPC measures, the management of linen plays an integral role in breaking the chain of infection, and the generic definition was coined by Buse et al. in 2018:

*“‘Laundry’ is often used as a generic term, but in fact it encompasses different categories – linen (sheets, towels, and bedding), outer clothing, and underwear, which have different implications for embodied practice, routines of washing, meanings of privacy, and identity”* [2]

Care home residents often have compromised immune systems, chronic illnesses, or disabilities, making them particularly susceptible to infection [3]. Within these settings, the risks associated with managed textiles, which will include residents’ bed linen, personal items of clothing, plus any reusable healthcare textiles such as hoists, need to be appropriately managed to reduce the risks of infection.

A range of microorganisms have been implicated in infectious disease outbreaks linked to linen mismanagement. Studies have been undertaken on pathogens such as *Clostridioides difficile*, methicillin-resistant *Staphylococcus aureus* (MRSA), and various gastrointestinal bacteria within acute settings [1]. It is well documented that these bacteria can survive on textiles, even under suboptimal environmental conditions. For instance, *C. difficile* spores are known to withstand standard laundering processes if not adequately disinfected, and norovirus can persist on fabrics and contribute to rapid transmission in healthcare environments [4–6]. These three studies alone and that of Owen and Laird [1] highlight that those linens processed inadequately can act as fomites, contributing to the spread of these pathogens [7], and those textiles can harbour pathogens for extended periods of time [6,8]. Primarily, these studies were conducted within hospital settings; their collective findings are not easily translatable to care home settings. All textiles present a potential risk for the transmission of infections to vulnerable individuals and should be treated differently based on the type of setting. The presence of pathogens on bedding, uniforms, curtains, and other textiles in care homes can contribute to the spread of infections, emphasising the need for rigorous infection control practices in these settings.

The transmission pathways of infections in care homes are multifactorial, with good linen management, policies and practices playing a pivotal role in avoiding transmission and overall IPC principles. The handling of contaminated items by staff without appropriate personal protective equipment (PPE) or adherence to hand hygiene can lead to cross-contamination [9]. As care home residents often share common areas, contaminated linen may become a vector for outbreaks, highlighting the need for stringent laundering protocols. There is limited research on the infection risk from linen to staff working within care homes. One study, although conducted in 1994, did report a nosocomial outbreak of Salmonella gastroenteritis among laundry workers in a nursing home [10]. Such findings highlight the dual requirement to always protect staff and residents through proper laundry hygiene. Several staff working in care homes is likely to be handling soiled items, including laundry, which could be putting them and their residents at risk of infectious diseases.

Within care homes, outbreaks of infectious disease represent a significant and ongoing public health challenge, often exacerbated by the high vulnerability of residents [11]. Respiratory infections, including influenza and more recently COVID-19, have frequently caused severe morbidity and mortality in care home populations [11,12]. The COVID-19 pandemic highlighted the susceptibility of care homes to outbreaks [12]. This was noted in studies conducted where rapid person-to-person transmission was observed due to close contact and shared spaces, with SARS-CoV-2 surviving on textiles for up to 72 hours [13]. The Department of Health and Social Care guidance [14] applies to any social care provision, highlighting that inadequate infection prevention measures, such as improper handling of soiled linen, have been identified as contributing factors in outbreak scenarios. To aid understanding, the following definitions were developed for used and infectious linen:

- Used linen: Linen that has been used but is not visibly contaminated with blood, urine, faeces, or vomit, and has not been in contact with a person known or suspected to be infectious.
- Infectious linen: Linen that has been used by a person known or suspected to be infectious and/or linen that is visibly contaminated (The terms fouled and soiled will no longer be used) with blood, urine, faeces, or vomit [15].

In the UK IPC guidance and policies exist such as; linen processing within adult social care: information sheet [16], which gives a link to “Prevention and control of infection in care homes as an information resource”; Health Technical Memorandum (HTM1–04) “Decontamination of linen for health and social care [17], which gives guidance on management and provision, engineering, equipment and validation, social care, and guidance for linen processors implementing BS EN 14065:2016 and the Safe Management of linen including uniforms and work wear. The accessibility, usability, and implementation of these policies within care home settings are not widely known, nor is it clear how they are implemented and monitored.

In the UK, the NHS also provides comprehensive guidelines for hospitals on environmental cleaning and laundry management as part of broader infection prevention strategies, recommending clear segregation between clean and soiled linens, adequate laundering temperatures, and the use of appropriate detergents [7]. The UK Health Security Agency (UKHSA) further highlights the necessity for care homes to develop risk assessments for infection control, including laundry procedures.

The current NHS guidance and evidence-based best practice emphasises the effectiveness of standardised linen management protocols in mitigating infection risks, which should be followed within all health and social care settings, including care homes. Despite the important role that correct and effective linen management plays within health and care settings, the extent and effectiveness of this across the UK care home sector are inadequately evidenced.

The Care Quality Commission (CQC) is an independent regulator of health and adult social care in England. Through their regular inspections and emphasis on IPC policies that are specific to the operational context of each care home [18].

Regardless of the quality and/or availability of IPC and Department of Health and Social Care (DoH) linen management guidelines little is understood about the effective implementation of the guidance criteria across the sector, there is an acknowledgement that resource constraints, variability in staff training and high staff turnover, and a lack of standardised auditing mechanisms may play a factor for lack of adherence as shown in studies looking at incontinence for example‌‌ [19].

Addressing and understanding the challenges for the care home sector requires a multi-faceted approach involving stakeholder collaboration, investment in infrastructure, and continuous professional development (CPD) across the care home workforce.

This study aims to explore care‑home staff and managers’ awareness and knowledge of current IPC policies and their implementation, and examines current linen‑management practices within care homes, including overall attitudes where applicable. The analysis considers implementation strategies, as well as the barriers and facilitators influencing effective IPC‑related laundry practice. This has led to the development of adapted national guidance for the safe management of linen within care homes. This incorporates evidence from key regulatory bodies, best practice guidelines, and studies undertaken in this field.

## Materials and methods

This study took a mixed-methods approach using online surveys, focus groups, and one-to-one interviews with staff working in care homes who are required to know and apply the latest IPC guidance. Data was collected from single and multi-site care homes across the UK with a recruitment period from June 2023 to November 2024. Initial findings from the online surveys were used to inform the qualitative aspect of the study. The research team conducted an independent analysis followed by a discursive analysis approach, which allowed for iterative development of themes, improving the rigour of the work through intercoder reliability.

### Ethics statement

The study received ethical approval from De Montfort University’s Health and Life Sciences Research Ethics Committee (Ethics number: 462424) and adhered to the British Education Research Association (BERA) ethical code of practice. All participants were given participant information sheets, informed consent for online questionnaires was inferred via completion of the survey, which was outlined in the participant information sheet, and written consent was obtained for interviews and focus groups.

### Data collection tools

#### Online surveys.

Four online surveys were created using Qualtrics, using both quantitative and qualitative questions, and some characteristics of the survey participants were included. This included their role and length of service in the care home sector. Surveys were distributed across social media and promoted by Knowlex (IPC conference provider), Textile Services Association (TSA), Infection Prevention Society (IPS), and ENRICH Scotland via their distribution lists.

Surveys one and two were directed at anyone accessing and managing infection control guidance, with survey one aimed towards staff working within the care home setting meeting this remit and survey two was only for care home managers or deputies with overall responsibility for IPC adherence. This allowed a wide range of views and experiences from across the sector and different types of aged care homes.

A third and fourth online survey was developed and distributed by the IPS care home special interest group (SIG) with a focus on the IPC standards for linen management to the same population groups as surveys one and two.

#### Sampling and recruitment strategy.

Survey: The proposed sampling strategy draws on distribution through nationally recognised and trusted bodies with established reach into the UK care home sector. By utilising these networks (Knowlex, TSA, IPS and ENRICH Scotland), the survey is disseminated via credible professional associations that maintain extensive membership and communication channels with care homes across the UK. This approach enhances the likelihood of achieving a broad, representative sample by engaging organisations that are well‑embedded in sector‑wide practice, quality improvement activity, and professional development. While this constitutes a form of opportunistic sampling, the use of reputable national bodies helps to strengthen reach, trust, and engagement across diverse care settings.

Focus Groups and Interviews: Participants were purposively invited to ensure representation from those with direct responsibility for IPC within the care home sector. This included care home managers and staff involved in developing, implementing, or overseeing IPC policies and procedures. To capture variation in organisational structure and operational context, invitations were extended to representatives from both single‑site care homes and larger multi‑site care home groups. As a qualitative arm to this study, no sample size was determined.

#### Qualitative approach.

Initial findings and themes elicited from the online surveys were used to inform the qualitative aspect of the study, and an outline study topic guide was developed. Care home managers were invited to take part in either a one-to-one interview or a focus group interview, depending on their preferences and availability. A total of 10 individuals consented to either a focus group or a one-to-one interview, and all interviews were digitally audio recorded.

The interviews delved further, using the survey results, to understand current IPC policies, barriers and challenges in implementing and managing linen processes.

### Data analysis

#### Quantitative data.

Descriptive statistics were used to summarise survey responses, including proportions and frequencies for variables. Differences in proportions between groups (e.g., staff vs. managers; single‑site vs. multi‑site homes) were assessed using two‑proportion z‑tests. Statistical significance was set at p ≤ 0.001.

#### Qualitative data.

Responses from the four online surveys and the interviews were gathered and grouped into individual themes using thematic analysis [20,21]. The data analysis using Braun and Clark’s [20,21] approach provided a more in-depth understanding of the application of current linen management within UK care home settings, looking at the adapted IPC guidance for care homes. A six-step process was followed: ‘familiarising yourself with your data’, ‘generating initial codes’, ‘searching for themes’, ‘reviewing themes’, ‘defining and naming themes’, and ‘producing the report’.

## Results

### Online surveys

In total, 1062 responses were received across all four surveys administered. Quantitative data collected using Qualtrics was used to perform basic calculations to give a percentage for each question.

#### Survey 1(care staff) & 2 (care home managers).

The first and second surveys in total attracted 901 responses; 406 from care staff (survey 1) and a further 495 of these responses were from care home managers (survey 2) employed in a variety of care homes in the UK. A wide cross-section of staff completed survey 1, including care assistants, registered nurses and nursing assistants, domestic staff, and those identified with specific roles such as surgical assistant, rehabilitation counsellor, and medical worker.

Of the 901 responses, 10% had worked in the sector for less than a year, 39% had worked in the care home sector for between 1–3 years, 38% between 4 and 10 years, and a further 13% for over 10 years. Indicating the longevity of service in the sector.

#### Where do care homes launder their linen?.

The majority of responses from care home managers indicated they had on-site laundering provision (82%), and over 90% of those indicated the use of commercial laundry machines as opposed to domestic machines bought on the high street. Care homes that did not have on-site facilities contracted external commercial operators. However, some responses indicated that they use high street coin-operated launderettes for all their laundry needs, which was surprising finding. When care home managers were asked if they were building a new facility, would they operate an On-Premise Laundry (OPL) or outsource to a commercial laundry, 46% of the multisite managers and 37% of single-site managers stated they would outsource.

#### Care homes with employed laundry staff.

For care homes with in-house provision, over 96% of care home managers indicated that staff were employed to work in this specific role, which included washing and ironing residents’ personal clothing items. There was no significant difference (p ≥ 0.001) between responses from single-site and multi-site managers.

Care home staff (65 respondents) who answered ‘no’ or ‘not sure’ to whether they had a dedicated laundry operative in their care home, were then asked if they had ever worked in their care home laundry facility, There was an almost even split of yes/no responses, indicating that, in addition to trained laundry staff, other staff within the home were also engaged in laundry practices. This demonstrates a significant difference in (z = −5.14, p ≤ 0.001) the responses from managers to those of the care home staff working within the care home.

#### What types of linen are laundered in OPLs?.

To understand further the types of laundering services offered, 495 managers shared details about the type of linen management they are providing in-house, referred to as OPL. Bed linen was the most processed textile, then residents’ personal clothing (44%), followed by staff uniforms (34%), bed linen (24%), and other items such as bathing towels, kitchen linen, and catering clothes.

#### How well is linen being processed in OPLs?.

We were keen to understand the perceptions of care home staff about how well linen is processed in-house, with a particular focus on bed linen, residents’ clothing, and staff uniforms. Overall, there were mixed responses, with under 50% agreeing that these items were processed well in their setting. This is worrying, implying that the standard of laundering by those carrying out this service is not viewed highly.

#### How often is bed linen changed?.

Our survey responses indicate different approaches that exist for the routine, regular changing of residents’ bed linen. We were not reviewing these individual policies, but wanted to understand the regularity of routine bed linen changes, in relation to used and infectious linen changes.

Care home staff (30% (110)) reported they changed bed linen daily or as required (26% (92)). Interestingly, the responses for this question differed between staff and managers. Managers (no significant difference (p = ≥ 0.001) between single-site and multi-site managers) were significantly more likely than staff to report a weekly bed‑changing policy (z = −4.56, p ≤ 0.001) than care home staff. With, 53% of managers indicated that bedding was changed weekly, compared to 23% of care home staff. Other responses included 3 times a week, or other. Surprisingly, there is no specific requirement for a resident’s “bed changing policy” in the CQC framework of key lines of enquiry.

#### Awareness of laundry operating standards.

The survey was interested in understanding the current awareness of the operating standards required for laundry management by care home managers and those providing the services in-house. Over 58% of care home managers working in a single-site care home indicated knowledge of the standards, increasing to 73% of those working in multi-site provision. Whilst this was initially encouraging, many of the respondents, care home managers and associated staff, were not conversant with the latest HTM01–04 [17] decontamination of linen for health and social care. In addition, there was limited awareness of the IPC risk management standard (BS EN 14065:2016) [14] employed within accredited commercial laundries.

#### Internal procedures/standards and training in the care home.

We were keen to understand the current practices for training and education on linen management within the sector, which forms part of the CQC inspection criteria.

In asking the care homes about their policies, we received mixed responses. Only 46% of care home staff reported knowing about the implementation of IPC procedures for linen management within their setting, but were aware of informal means of communicating between staff about best practice. Less than 30% of all care home manager respondents stated they had a procedure for onboarding new staff to relevant laundry procedures and management. This lack of understanding and implementation of IPC policies indicates a reliance on informal means of communicating important IPC policies rather than any formal training, structured means of communicating changes, or important guidance on linen management.

Interestingly, when care home staff were asked if they had personally had any formal training on operating laundry, only 22 care workers responded, and 45% (10) of these respondents reported that they received formal laundry training when they were first employed. Half of which reported that the laundry machine(s) user guide(s) were shared with them when they were first employed. In contrast, 65% of managers believed that care home staff were being trained in laundry processes; this difference, however, was not statistically significant (z = −1.29, p ≥ 0.001).

There was a consensus between care home staff and managers that they would like more training/guidance on running OPLs; 86% of care workers and 98% of care home managers stated that they would; other responses included no or not sure.

### Infection control processes - survey 3 (care home staff) and survey 4 (care home managers)

The 3rd and 4th online surveys, which were directly related to the IPC protocols, had a total of 161 responses; 57 care home staff (survey 3) and 104 responses were from care home managers (survey 4).

Most care workers, of the care homes that had on site laundries (86) (84% (21), and managers, 87% (53), stated that there was a dirty-to-clean flow in the laundry room; this still leaves up to 16% (4) of care workers and 13% (8) managers surveyed stating they not follow this critical infection control principle. Most staff and managers stated that there is a designated area for the storage of clean linen, which is separate from used and infectious linen, with bags and trolleys mainly being used to transport linen around the care home.

The potential for manual sluicing of linen to be a cross-contamination route of infectious disease is high. Manual sluices being within a laundry is not advised by the DoH in their laundry IPC guidelines [7]. Of the 86 care homes with laundries on site and answering yes to manual sluicing practices, 44% (24/54) of managers and 44% (11/25) of care home staff respondents confirmed that they had a manual sluice facility/sluicing basin situated in the laundry room, contravening the DOH advice. Furthermore, of the 80 respondents who stated they used water-soluble bags for infected linen, only 68% (17) of care home staff and 67% (37) of managers stated that their full water-soluble bags for infected linen were stored in a solid lidded container/non-soluble plastic bag. This indicates that approximately 30% of respondents may have a contamination risk resulting from the inadequate storage of infected linen within their care homes.

### Qualitative findings

In conducting the analysis of the data from the focus groups and one-to-one interviews, we identified 4 themes: 1. Implementation of regulations, 2. The challenges and barriers to applying laundry regulations 3. Mixed linen items and adherence to the regulations 4. Supporting the sector with implementation, education, and training. These themes were developed from 10 participants (n = 4 from a focus group and n = 6 from individual interviews) with a broad understanding and application of laundry regulations and all work within the UK care home sector, as shown in Table 1.

**Table 1:** Study participants.

Data collection tool:	Participants:	Participant code:
Focus group	A catering and housekeeping support specialist based in one care home within a large multi-site care home company - primary role is to support managers in their homes with catering and laundry.	A
	An infection control and prevention specialist nurse for a large multi-site care home company - role is to support approximately 280 care homes across England, Scotland and Wales in IPC.	B
	A clinical improvement lead and nurse by background who specialises in human factors in patient safety.	C
	A technical consultant within the laundry industry, advising on engineering and hygiene control. Works within the care home sector to support hygiene control and laundry, and has worked in the textile services industry for several years.	D
One-to-one interviews	A care home manager of a large multi-site care home, with one multi-use in-house laundry, the care home comprises five units with a total of 86 beds	E
	A laundry manager in the same facility as participant E, worked for 29 years in the sector.	F
	Manager of a single-site care home with 20 beds	G
	Manager of a single site with 10 beds	H
	Regional director who manages five care homes – total beds 330	I
	Manager of a multi-site care home company that supports over 200 residents	J

#### 1. Implementation of regulations.

As expected, within the arena of linen management, there are some very specific guidelines. For example, HTM-01–04 and BS EN 14065 guidance [17], the latter relates to laundry risk management systems, such as those required within commercial laundry providers. Whilst the HTM-01–04 is often cited, it is written for use within a broad range of healthcare linen providers and advises on management, engineering and validation of healthcare laundries. The development of adapted IPC guidance for care homes transforms existing guidance into a more workable approach for linen management, and this was discussed in the interviews.

From our survey results, over 1,000 responses, there was an overall lack of knowledge about the IPC guidance in relation to linen and laundries. In our interviews, we wanted to explore some of the broad themes that emerged around barriers and how care homes might be adopting these regulations.

Overall, there was consensus that a lack of knowledge and awareness about laundering governance exists. Many of the participants declared they had not heard of either IPC policy (HTM-01–04 and BS EN 14065:2016) [17], as the following quote illustrates:

*“Care home workers are told what they need to do in terms of following the procedures, but we don’t mention the HTM with the training, the care workers are instructed on what to do to deliver the standards” (participant B)*

These two participants agreed that their role was to provide the care home managers with the information that they needed to implement, making it as easy as possible:

*“So, they don’t have to wade through HTM, they’ve got a team and me in my role to help interpret what’s in it to implement it” (participant A)*

In our interviews, we attracted a wide range of expertise, including those from multiple site care homes, regional care home managers, single site care homes, and experts in laundry management, in our focus group. It was noticeable that the difference in perception of larger and multiple-site care homes compared to single or smaller care homes to implement the guidance, for example:

*“A chain of care homes has lots of support, so they have a team that is dedicated to training and developing procedures and also work with procurement so there is dedicated laundry equipment, whereas smaller care homes may not have as much support” (participant B)*

Conversely, a care home manager of a small (20-bed) provider commented that:

*“The benefit of having a small organisation is that we can lead from the top; we’re fortunate that we’ve got a senior management structure. … I’m aware that some fellow managers have their own laundry team and industrial machines, and I can see the benefits of that. But I think the fact that we’ve got reduced numbers, [and] we adhere to the protocols, [it] works equally as well” (participant G)*

#### 2. The challenges and barriers to applying laundry guidance in care homes.

In the UK, there are over 17,000 independent care home providers [22]. Some are purpose-built, but many are not and often repurposed from homes, hotels, and the like. Many converted buildings may be inadequate to meet the IPC guidance, such as guidance for linen segregation.

When probing any observations made about implementing to meet the requirements for hygiene and overall managing linen, there was an acknowledgement that many homes are not purpose-built and therefore may struggle to contain and consistently meet the IPC guidance:

*“Care homes with a smaller laundry room will find it more challenging to segregate clean and dirty linen. If there’s a care home with a smaller laundry room, it’s more difficult to apply the processes, no matter how good they are. Care homes in older buildings need more input” (participant B)*

This was described with a single-site, 10-bed care home:

*“Mostly it’s the space, we wouldn’t have space for that really. It’s quite a cramped laundry room. I think that potentially the only one [(barrier)] though” (participant H)*

There was agreement that the lack of limited space available in care homes does present some challenges to meet the requirements of the IPC guidance:

*“I think what people fail to recognise is that care homes are homes, not hospitals, so you have to accept that there are some differing risks and challenges as opposed to a hospital environment. We don’t have as big a set-up for laundry facilities; we have 2 washers and 2 dryers. They’re industrial, they’re not domestic, but that still means that they’re running 24 hours a day” (participant I)*

Limited space, as well as cost implications, were also challenges raised:

*“We’ve talked about industrial machinery, and there are a couple of hurdles with that for us. They’re never the right size to fit in a domestic spacer, and that’s a bit of a challenge and also a cost. We’re a charity and [there are] cost implications” (participant I)*

The challenge, of course, is for care homes that operate in-house facilities when this malfunction:

*“If [the machines] break down, we don’t have a backup system. So, we have to make sure that we have contractors who can come out as soon as possible. That was one of the biggest challenges in COVID: getting people to come out when they break down. And then where do we go if that happens, because we don’t want cross–infection” (participant I)*

To provide segregation of resident items, rather than segregation of used and infected items as deemed in the guidance, the participant from one small single 10-bedded care home was able to adopt a workable routine with their residents to overcome the challenges of minimum space and use of a domestic appliance:

*“Because it is a small care home, residents can each have their own washing days, because they have their own days, it doesn’t tend to be waiting around, it all just goes in straight away and gets cleaned” (participant H)*

Another participant shared a common issue when using external, agency staff:

*“The problem we tend to find is that we have a lot of agency staff, and it’s very difficult to give the necessary information with regards to segregation of laundry, some agency staff just throw everything in the [laundry] bag … so sometimes they’ll put soiled pants in red bags” (participant A)*

When asked about infection prevention policies in their care homes, they recognised the complexity of making the guidance easy to access and understood by those responsible for this aspect of the home:

*“Written policies for infection prevention and control and COVID, and tried to make them straightforward [and] user-friendly. Rather than 100 pages that no one ever absorbs” (participant J)*

When participants were asked if they were aware of any specific linen management policies for IPC. Many participants were either not aware of such polices or, if they were, could not easily understand how this translated to actual practice:

*“It is covered by the two infection control policies, I think. Some of the challenge is more about knowing what to do, than actually doing it” (participant J)*

#### 3. Mixed laundry items and adherence to the guidance.

One of the concerns raised by the care homes across the four surveys was knowing how to process a variety of textiles. This is often related to the laundering of residents’ clothing. This is also an aspect most often raised by relatives as a reason why they take items home to be laundered:

*“…. if things go wrong in the laundry, it’s probably the highest number of complaints any care home gets” (participant I)*

For staff dealing with a wide variety of textiles, this can present additional workload pressures. For example, if residents were to soil themselves:

*“The staff sometimes just put all of the laundry, including residents’ clothes and throw everything into the same wash, and then they’re washed in the sluice wash, so all their [items] come back ruined”, causing the residents to complain about the condition and damage of their clothes (participant A)*

The challenge of laundering residents’ personal wear was noted by another participant:

*“It is not suitable to wash residents’ clothing at 70°C, as it can result in shrinking and ruining their clothes. There has to be a balance between the IPC and the fact that these are people’s belongings, and this is their home” (participant B)*

A manager of five care homes concurred with this view, noting the challenges compared to hospital settings, and the pressures from residents’ family members:

*“I think in care homes, a lot of our issues are around personal clothing. Which I don’t think is a problem as much in hospitals, because the [residents are] wearing their normal clothing … we have a lot of issues with that. From woollen cardigans to silk cardigans, all those kinds of things. We have more wash cycles as opposed to bed linen, towels, etc., and that can cause a lot more obstacles and challenges, a lot of pressure and expectation from family members to staff to make sure they’re laundered correctly and brought back correctly” (participant I)*

Care homes want to adhere to the IPC guidance, but this can conflict with dealing with the wishes of relatives, meeting residents’ needs and balancing the practicalities of providing a good laundry service in-house. There was general agreement that guidance around personal clothing and the IPC guidance wasn’t clear or well-known:

*“I think the only thing it says with people’s clothing [is] wash at the highest temperature clothes will withstand” (participant B)*

*“One of the biggest [challenges], to do a hot wash, you’re killing people’s clothes and personal items (participant J)*

#### 4. Supporting the sector with implementation, education, and training.

There was consensus in the focus group and interviews that everyone working in the sector wanted to improve and provide the best possible service for their residents and a safe working environment for everyone, including the correct management of linen.

One clear aspect was how to improve training, not just the knowledge and education around the IPC guidance. This was emphasised when discussing the errors made by staff:

*“A lot is human error, as opposed to following the policies and procedures” (participant A)*

The challenge of onboarding agency staff with their linen management procedures was a concern:

*“In some homes we do struggle with [agency staff], and the problem is you can’t train them due to the frequency or infrequency in which they work within the care home” (participant A)*

Likewise, a specialist infection control nurse commented:

*“[We] invest so much time in training and bombard them [agency staff] with information on induction, and then 6 months later they go and work elsewhere” (participant B)*

Despite the challenges for linen management, some care homes were working on developing forms of staff training and resources to aid their staff:

*“We’ve done easy-reads, as much as we can to educate … It proves a challenge, it’s something, I think, as an organisation we’ve done well, we’ve done it how we’ve educated and communicated everything, and I think that’s what’s key in most of these situations. When people know what they’ve got to do and how they’ve got to do it, you’re 80% there” (participant J)*

There was wide acknowledgement that having training material to support the IPC implementation would have an impact moving forward, removing some of the barriers and challenges which care homes and their staff face:

*“A pack that signposts them to guidance documents, websites and resources. If I were a home manager, that would be really useful” (participant B)*

*“It would depend on how it’s presented [resources], if it was complicated and inaccessible, then no, but if it’s easy to understand, then yes” (participant E)*

Further highlighting the importance of explaining the reasons for following such guidance:

*“[It’s] Incredibly important, because if people understand why they’re doing it and the purpose and benefit of it, they’re much more likely to do it than if I tell them to do it and it feels like extra work for no reason” (participant F)*

*“It’s something I would really welcome. Because it’s not only about ticking the box from an inspector’s interview point of view. Our fundamental responsibility is to keep everyone safe, and any tool that enables us to do that and do better, I’d embrace it wholeheartedly” (participant G)*

Those involved in the interviews further explained how they felt that infection control guidance for linen management was perhaps not as strong as other forms:

*“It’s ironic because we have infection control training, we’ll look at different aspects of that, we look at food hygiene, have food hygiene certificates, but when it comes to linen, I mean really the sort of potential for contamination and cross infection … it outweighs the other areas where we get really robust training, so I think yeah, you’ve identified a need, definitely” (participant G)*

A further aspect that was explored in the surveys related to residents’ bed linen. There were differing views and policies in place, with no consensus. Each home operated its in-house approach. Some had a targeted approach to checking beds daily and changing if required, whilst others operated a routine of bed linen changed every other day, regardless of soiling or not.

One specialist nurse for a large chain noted they were not aware of a policy for bed changing:

*“At each home they manage it – on a national company level, I don’t think we specify, I may be wrong, but I haven’t seen it” (participant B)*

And clarified it may be something that would be useful:

*“I think it would be, because [with] hospitals … there was always a debate as to how frequently to change sheets, and whether you needed to change sheets every day? So, if there is some evidence and guidance for care homes that would be [useful]” (participant B)*

Whilst training materials and resources for implementation of the IPC guidance were generally welcomed, the input from local authorities and CQC was also valued:

*“[We] get support from local authorities that do annual audits and inspections, and they give us advice and updates from there, but they don’t always give you solutions, just give you actions” (participant I)*

This study provides a distinctive and substantive contribution to understanding linen management and IPC within the care home sector. As one of the most extensive investigations undertaken in this context, it offers a comprehensive examination of current linen management practices and sheds light on how national guidance is interpreted, operationalised, and enacted by those responsible for its implementation.

### Development of adapted management of Linen guidance for use in the care home setting

The analysis of the data from both the quantitative and qualitative approaches informed the development of an adapted care home linen management guidance with the formation. A national IPC laundry management group was established through the Infection Prevention Society Care Home Special Interest group (SIG) collaborators.

#### Co-creation.

To develop relevant and meaningful guidance, both in terms of scientific accuracy and usability within the care home setting, it was of vital importance that the linen management guidance was written alongside a range of collaborators, each with different skill sets and expertise. By merging the key science, practice, and education principles within the co-creation process, the team was able to develop meaningful guidance that could be easily interpreted and used in a diverse range of care home environments. Co-Creation has become a widely recognised approach to pedagogic practice as a means of actively involving individuals or groups of people in areas such as guidance development, and this approach has links with various educational theorists, including the seminal works by Dewey [23] and Hooks [24].

#### Collaborators.

Education specialists, scientists (microbiology and chemistry), nursing practitioners with expertise in the NHS and care home settings, NHS Trusts, local authorities, independent care homes, health protection, county councils, and the UKHSA.

#### Guidance development approach.

When creating the education and training guidance, for it to be followed effectively, it is fundamental that the guidance states not only how to complete certain tasks, but also why it is important to complete them. Psychologists highlight that it is the motivational aspect of the ‘why’ that encourages people to perform certain tasks or engage in behaviours [25]. With specific reference to this guidance, the ‘why’ is integral because it enables the care home employees to understand the reason for their training, ultimately leading to improved performance and engagement with IPC around linen management.

The developmental approach to constructing the guidance was to follow a risk‑management approach, drawing directly on the established methods used by one of the project collaborators, an experienced IPC trainer and risk manager whose standardised framework guided the structure and prioritisation within the adapted guidance, recognising the diverse capabilities of care homes in adhering to guidance. A risk-based framework was applied, outlining requirements, their rationale, and a risk assessment tool to document non-compliance and mitigation measures was included with the guidance for use by care homes. Uniquely, this guidance prioritises training and education at the outset, emphasising their critical role in the successful implementation of the adapted laundry guidance. The methodology then follows a logical sequence, tracking linen from its ‘clean’ state through use, contamination, processing, and return to cleanliness within the laundering cycle.

### Linking empirical findings to the development of the adapted IPC Linen‑management guidance

The new guidance, ‘Safe Management of Linen in Residential, Nursing or other Social Healthcare Environments’, was developed to provide safe, workable laundering practices for the care home setting. The full IPC guidance can be located at Infection Prevention Society - Safe Management of Linen in the Health Care Environment | IPS, Skills for Care - GO Online: Inspection Toolkit – search for laundry.

To ensure that the adapted linen‑management guidance reflected both the evidence base and the operational realities of care homes, the IPS Care Home SIG used a structured decision‑making process. The SIG reviewed all themes emerging from the surveys, focus group, and interviews, mapping them against the key principles of existing regulatory frameworks, particularly HTM 01‑04, and prioritising those findings that had direct implications for safe, feasible laundering practice within the care‑home environment. Themes were only included if they met two criteria: (1) they were consistently identified across at least one qualitative and one quantitative source, and (2) they aligned with the risk‑management, safety, and operational principles set out in HTM 01‑04.

The included findings reflected the strongest cross‑data themes. Survey responses highlighted inconsistent implementation of IPC laundry procedures and uncertainty about correct linen segregation, while both interview and focus‑group participants emphasised a widespread need for further, ongoing education. Participants also described barriers such as limited facilities, unclear roles, and the routine challenges of communal living settings. These findings collectively informed decisions about which elements required explicit instruction, clearer justification, or additional tools within the guidance.

These results directly shaped the development approach. The SIG then applied a risk‑management framework to each included theme, using HTM 01‑04 principles to structure the requirements, their rationale, and associated mitigation measures. The sequence of the guidance tracking linen from clean state linen, through use, contamination, laundering, and return to clean state was also informed by data showing that staff often understood individual tasks but not the full workflow. Thus, the final adapted guidance reflects both the evidence generated through the study and the core principles of care‑home IPC, ensuring that staff have clarity on what to do, how to do it, and why efficient, best laundering practice reduces the transmission of infection.

## Discussion

The imperative for comprehensive and robust linen-management policies within IPC frameworks in care homes cannot be overstated. The profound impact of the COVID-19 pandemic on UK care homes underscored the necessity of understanding, implementing, and monitoring effective IPC measures across all operational domains, including laundering processes, to mitigate the transmission of infectious diseases [13,26,27]. Evidence from Bockmühl et al. [26] demonstrates the persistence of microorganisms within washing machines; although their study focused on domestic settings, the findings are directly pertinent to care homes, where domestic-grade laundry equipment remains widely used. This research highlights substantial risks associated with inadequate laundering systems and reinforces the need for stringent, evidence-informed IPC measures related to textile management in these environments. Despite its centrality to maintaining safe care, linen processing remains an underexamined component of IPC in residential care home settings where residents face elevated vulnerability due to age-related comorbidities, immune system decline, and the inherent risks associated with shared living spaces [28].

Further concern is raised by Cayrou [29], who found that 50% of domestic washing machines failed to reach disinfection temperatures, with all machines harbouring antibiotic-resistant microorganisms. This evidence makes it clear that care homes must *not* rely on domestic appliances. Instead, they must use commercial laundry equipment that is fit for purpose, correctly installed and serviced/maintained by a professional organisation to safeguard the health of residents and staff working in these environments. Should care homes not be able to meet these stringent requirements, then outsourcing to commercial laundries that are BS EN 14065:2021 accredited should be considered.

The qualitative findings illustrate the complexity of linen management across a variety of care homes. The findings demonstrate that whilst all care homes and staff involved in the study had some awareness of IPC for linen management, many of the respondents concurred that they were unable to easily translate this into operational running of their own care home laundry. This highlights further the practical issues for care homes in adhering to HTM1:04, which is not specific to care home settings but rather to a hospital setting.

Barriers relate to the built environment in which many care homes function; many are in converted buildings or domestic-scale facilities that cannot accommodate dirty-to-clean workflow zoning [17,30]. This operational reality underscores the need for sector-specific adaptations of IPC protocols that reflect the structural and staffing constraints of care homes. Laundering residents’ personal clothing items is also a problematic area for care homes and is a repeated issue raised by relatives.

Our study did not explore how residents’ clothing was handled when using external contractors, and this is an area for further exploration. Care homes may also wish to outsource for commercial or operational reasons, particularly as the UK is seeing bespoke care homes with large-scale laundries that offer all textile care, including personal clothing.

Prevention and control of infection in care homes – an information resource [17] states under the description of bedding that “linen should be changed at frequent intervals and when soiled”. This area could be explored further to improve guidance and consistency given for care homes.

Ultimately, effective and well-managed laundering processes are not optional; they are a vital component in controlling the presence and spread of pathogenic and odour-causing microorganisms [28]. Minimising the risk of cross-contamination of linen must be treated as a core element of infection prevention strategies in care home environments.

Our qualitative findings highlight a knowledge-practice gap, with care staff expressing an understanding of IPC principles but struggling to apply them within their specific laundry environments, a challenge supported by prior research on IPC implementation barriers in social care settings [31]. Findings from this review [31] strongly indicate that more accessible guidance for dealing with infection control and operationalising the current guidance within care homes is urgently required.

A key element raised during the qualitative arm of this study was implementing a clear and defined process for the variety and scale of textiles that care homes handle daily. This includes residents’ clothing and personal items that may require different cleaning regimes. Additionally, all participants agreed that simplified guidance, applicable to care home settings, not just clinical settings, would be welcomed.

As a result of this work, a linen management working group was formed at the IPS care home SIG to develop accessible best-practice guidance, education and training documentation.

The following recommendations were included in the adapted linen management guidance for care homes, with further work ongoing to develop training videos and bite-sized learning:

**Simplified interpretations of HTM1:04:** Creating a simplified package of guidance that clearly illustrates the HTM1:04 policy so that they are understandable, therefore, more likely to be followed. **Multi-format training materials:** Develop different styles of learning material, for example, 5–10-minute bite-sized videos, user-friendly paper guidance, selected posters (though not too many) with important key messages. **A risk assessment toolkit:** To support care homes in understanding the risks and “dos and don’ts” when laundering different items. Examples of good and bad practice should be included. **Template policies and procedures:** That care homes can incorporate into their own individualised training programmes, and finally, **Clear explanations of why each protocol matters:** Guidance needs to explain what to do, but also why it is important.

These initiatives reflect best practices in knowledge translation and have the potential to support meaningful, sustainable improvements in IPC compliance across the sector [32].

## Conclusions

Effective linen management constitutes a critical, yet frequently under-recognised, component of broader IPC practices within care homes. Findings from our large-scale mixed-methods study underscore the extent of this oversight and highlight the implications for resident safety and organisational resilience. As demographic shifts drive increasing demand for long-term care provision, the need for timely, practical, and evidence-informed guidance has become increasingly urgent. The UK care home sector continues to evolve in response to changing demographic, regulatory, and policy landscapes; accordingly, linen management guidance must be responsive to these developments. It is incumbent upon stakeholders to ensure the availability of clear, actionable, and contextually relevant resources that align with the realities of care home practice and support the consistent application of IPC standards.

It is incumbent on those working in this field to be cognisant of the effective approaches that can be adhered to ensure the safety and awareness of infection control measures for all those working or encountering linen items. The current guidance, developed for application in hospitals, is not easily transferable or relatable to the variety of care home settings, often not in purpose-built environments. More relatable guidance is required that is adapted for the care home sector and is fit for purpose and application by those providing linen management to meet IPC standards.

Future research should prioritise the development of cost-effective and scalable strategies that can be integrated seamlessly into the routine operations of busy care home environments. As scientific and technological innovations continue to progress, considerable opportunities exist to enhance laundry-related IPC practices. Such advancements hold the potential to strengthen organisational safety, support staff in delivering high-quality care, and improve outcomes for some of the most vulnerable individuals within our communities.

## References

[1] Owen L, Laird K. The role of textiles as fomites in the healthcare environment: a review of the infection control risk. PeerJ. 2020;8:e9790. doi: 10.7717/peerj.9790 32904371 PMC7453921

[2] Buse D, Twigg J, Nettleton S, Martin D. Dirty linen, liminal spaces, and later life: meanings of laundry in care home design and practice. Sociol Res Online. 2018;23(4):711–27.

[3] Goodyear N, Markkanen P, Beato-Melendez C, Mohamed H, Gore R, Galligan C, et al. Cleaning and disinfection in home care: a comparison of 2 commercial products with potentially different consequences for respiratory health. Am J Infect Control. 2018;46(4):410–6. doi: 10.1016/j.ajic.2017.09.033 29169933

[4] Tarrant J, Jenkins RO, Laird KT. From ward to washer: the survival of Clostridium difficile spores on hospital bed sheets through a commercial UK NHS healthcare laundry process. Infect Control Hosp Epidemiol. 2018;39(12):1406–11. doi: 10.1017/ice.2018.255 30322417

[5] Fijan S, Sostar-Turk S, Cencic A. Implementing hygiene monitoring systems in hospital laundries in order to reduce microbial contamination of hospital textiles. J Hosp Infect. 2005;61(1):30–8. doi: 10.1016/j.jhin.2005.02.005 15975691

[6] Fijan S, Pahor D, Turk S. Survival of Enterococcus faecium, Staphylococcus aureus and Pseudomonas aeruginosa on cotton. Text Res J. 2017;87(14):1711–21.

[7] NHS England. National infection prevention and control manual (NIPCM) for England. 2025. [cited 2025 Apr 2]. Available from: www.england.nhs.uk/national-infection-prevention-and-control-manual-nipcm-for-england/chapter-1-standard-infection-control-precautions-sicps/

[8] Riley K, Williams J, Owen L, Shen J, Davies A, Laird K. The effect of low-temperature laundering and detergents on the survival of Escherichia coli and Staphylococcus aureus on textiles used in healthcare uniforms. J Appl Microbiol. 2017;123(1):280–6. doi: 10.1111/jam.13485 28489297

[9] Curryer C, Russo PL, Kiernan M, Wares KD, Smith K, Mitchell BG. Environmental hygiene, knowledge and cleaning practice: a phenomenological study of nurses and midwives during COVID-19. Am J Infect Control. 2021;49(9):1123–8. doi: 10.1016/j.ajic.2021.04.080 33915230 PMC8074523

[10] Standaert SM, Hutcheson RH, Schaffner W. Nosocomial transmission of Salmonella gastroenteritis to laundry workers in a nursing home. Infect Control Hosp Epidemiol. 1994;15(1):22–6. doi: 10.1086/646813 8133005

[11] Kung P-J, Fang C-J, Cheng Y-Y, Chen C-M. Health and care workers in long-term care facilities and their role in preventing emerging infectious diseases: a scoping review. J Nurs Scholarsh. 2024;56(2):260–81. doi: 10.1111/jnu.12936 37853997

[12] Gordon AL, Goodman C, Achterberg W, Barker RO, Burns E, Hanratty B, et al. Commentary: COVID in care homes-challenges and dilemmas in healthcare delivery. Age Ageing. 2020;49(5):701–5. doi: 10.1093/ageing/afaa113 32402088 PMC7239229

[13] Owen L, Shivkumar M, Laird K. The stability of model human coronaviruses on textiles in the environment and during health care laundering. mSphere. 2021;6(2):e00316-21. doi: 10.1128/mSphere.00316-21 33910996 PMC8092140

[14] Department of Health and Social Care. Infection prevention and control in adult social care settings. 2022. [cited 2024 Nov 20].Available from: https://www.gov.uk/government/publications/infection-prevention-and-control-in-adult-social-care-settings

[15] Laird K, McMahon J, Hook J, Allen J. Guidance for the Safe Management of Linen in Residential, Nursing or Other Social Healthcare Environments. 2024. https://tsa-uk.org/wp-content/uploads/Guidance-for-the-safe-management-of-linen-161024.pdf

[16] UK Health Security Agency (UKHSA). Linen processing within adult social care: information sheet. 2023.[cited 2025 May 2]. Available from: https://www.gov.uk/government/publications/linen-processing-within-adult-social-care-information-sheet

[17] NHS England. Guidance for linen processors implementing BS EN 14065, Health Technical Memorandum 01-04: Decontamination of linen for health and social care. Guidance for linen processors implementing BS EN 14065. 2016. [cited 2023 Dec 6]. Available from: www.england.nhs.uk/wp-content/uploads/2021/05/BS_EN14065.pdf

[18] Care Quality Commission (CQC). Infection prevention and control in care homes. 2022. [cite 2024 Oct 18]. Available from: www.cqc.org.uk/guidance-providers/residential-adult-social-care/infection-prevention-control-care-homes

[19] Flanagan L, Roe B, Jack B, Shaw C, Williams KS, Chung A, et al. Factors with the management of incontinence and promotion of continence in older people in care homes. J Adv Nurs. 2014;70(3):476–96. doi: 10.1111/jan.12220 23889325

[20] Braun V, Clarke V. Using thematic analysis in psychology. Qual Res Psychol. 2006;3(2):77–101.

[21] Clarke V, Braun V. Thematic analysis. J Posit Psychol. 2017;12:3, 297–8.

[22] Nuffield Trust. What does the provider market look like across the four countries? 2023. [cited 2025 Mar 23]. Available from: www.nuffieldtrust.org.uk/news-item/what-does-the-provider-market-look-like-across-the-four-countries

[23] Dewey J. Democracy and Education: An Introduction to the Philosophy of Education. New York: The Macmillan Company; 1916.

[24] Hooks B. Teaching to transgress: education as the practice of freedom. New York: Routledge; 1994.

[25] Filgona J, Sakiyo J, Gwany D, Okoronka A. Motivation in learning. AJESS. 2020;10(4):16–37.

[26] Bockmühl DP, Schages J, Rehberg L. Laundry and textile hygiene in healthcare and beyond. Microb Cell. 2019;6(7):299–306. doi: 10.15698/mic2019.07.682 31294042 PMC6600116

[27] Decker B. The Role of health care laundry in infection prevention. ICT. 2022;26(6):16–8.

[28] Abney SE, Ijaz MK, McKinney J, Gerba CP. Laundry hygiene and odor control: state of the science. Appl Environ Microbiol. 2021;87(14):e0300220. doi: 10.1128/AEM.03002-20 33962979 PMC8231443

[29] Cayrou C, Silver K, Owen L, Dunlop J, Laird K. Domestic laundering of healthcare textiles: disinfection efficacy and risks of antibiotic resistance transmission. PLoS One. 2025;20(4):e0321467. doi: 10.1371/journal.pone.0321467 40305442 PMC12043170

[30] Health and Safety Executive (HSE). Health and safety in care homes. 2023. [cited 2025 Apr 29]https://www.hse.gov.uk/pubns/books/hsg220.htm

[31] Houghton C, Meskell P, Delaney H, Smalle M, Glenton C, Booth A, et al. Barriers and facilitators to healthcare workers’ adherence with infection prevention and control (IPC) guidelines for respiratory infectious diseases: a rapid qualitative evidence synthesis. Cochrane Database Syst Rev. 2020;4(4):CD013582. doi: 10.1002/14651858.CD013582 32315451 PMC7173761

[32] Grol R, Grimshaw J. From best evidence to best practice: effective implementation of change in patients’ care. Lancet. 2003;362(9391):1225–30. doi: 10.1016/S0140-6736(03)14546-1 14568747

